# The Genetic Landscape of Patent Foramen Ovale: A Systematic Review

**DOI:** 10.3390/genes12121953

**Published:** 2021-12-06

**Authors:** Matteo Paolucci, Chiara Vincenzi, Michele Romoli, Giulia Amico, Isabella Ceccherini, Simona Lattanzi, Anna Bersano, Marco Longoni, Simona Sacco, Fabrizio Vernieri, Rosario Pascarella, Franco Valzania, Marialuisa Zedde

**Affiliations:** 1 Headache and Neurosonology Unit, Neurology, Campus Bio-Medico University Hospital, Via Alvaro del Portillo, 200, 00128 Rome, Italy; f.vernieri@unicampus.it; 2 Neurology Unit, “M. Bufalini” Hospital, AUSL Romagna, Viale Giovanni Ghirotti, 286, 47521 Cesena, Italy; michele.romoli@auslromagna.it (M.R.); marco.longoni@auslromagna.it (M.L.); 3 Neurology Unit, Stroke Unit, AUSL-IRCCS di Reggio Emilia, via Amendola 2, 42122 Reggio Emilia, Italy; chiaravincenzi85@gmail.com (C.V.); franco.valzania@ausl.re.it (F.V.); marialuisa.zedde@gmail.com (M.Z.); 4 Laboratory of Genetics and Genomics of Rare Diseases, IRCCS Giannina Gaslini, Via Gerolamo Gaslini 5, 16147 Genova, Italy; giulia.amico87@gmail.com (G.A.); isa.c@unige.it (I.C.); 5 DINOGMI-Universite degli Studi di Genova, Largo P. Daneo,3, 16132 Genova, Italy; 6 Neurological Clinic, Department of Experimental and Clinical Medicine, Marche Polytechnic University, 60121 Ancona, Italy; alfierelattanzisimona@gmail.com; 7 Cerebrovascular Unit, Fondazione IRCCS Istituto Neurologico Carlo Besta, 20133 Milan, Italy; anna.bersano@istituto-besta.it; 8 Neuroscience Section, Department of Biotechnological and Applied Clinical Sciences, University of L’Aquila, 67100 L’Aquila, Italy; simona.sacco@univaq.it; 9 Neuroradiology Unit, AUSL-IRCCS di Reggio Emilia, via Amendola 2, 42122 Reggio Emilia, Italy; rosario.pascarella@ausl.re.it

**Keywords:** Congenital Heart Defects, Atrial Septal Defects, paradoxical embolism

## Abstract

Patent Foramen Ovale (PFO) is a common postnatal defect of cardiac atrial septation. A certain degree of familial aggregation has been reported. Animal studies suggest the involvement of the Notch pathway and other cardiac transcription factors (GATA4, TBX20, NKX2-5) in Foramen Ovale closure. This review evaluates the contribution of genetic alterations in PFO development. We systematically reviewed studies that assessed rare and common variants in subjects with PFO. The protocol was registered with PROSPERO and followed MOOSE guidelines. We systematically searched English studies reporting rates of variants in PFO subjects until the 30th of June 2021. Among 1231 studies, we included four studies: two of them assessed the *NKX2-5* gene, the remaining reported variants of chromosome 4q25 and the *GATA4* S377G variant, respectively. We did not find any variant associated with PFO, except for the rs2200733 variant of chromosome 4q25 in atrial fibrillation patients. Despite the scarceness of evidence so far, animal studies and other studies that did not fulfil the criteria to be included in the review indicate a robust genetic background in PFO. More research is needed on the genetic determinants of PFO.

## 1. Introduction

Paradoxical embolism through Patent Foramen Ovale (PFO) is an important cause of cryptogenic ischemic stroke, especially in younger patients [1]. Its diagnosis may be suspected based on Transcranial Doppler (TCD), a screening test achieving high sensitivity and specificity values (96.1% and 92.4%, respectively) in detecting right-to-left shunts [2]. PFO confirmation requires transesophageal echocardiography (TEE), the gold standard diagnostic test, or transthoracic echocardiography (TTE) with intravenous contrast agent. However, the latter is affected by a lower sensitivity value (47.5% for TTE with intravenous contrast) [2]. PFO is highly prevalent in the general population [3], ranging from 35% in younger subjects to 20% in the elderly [4]. Migraine with aura carries an even higher rate of PFO [5], particularly large ones [6]. In this condition, PFO shows a strong familial aggregation with an autosomal dominant inheritance pattern [7]. Consistent with this observation, the rate of PFO in siblings of young patients with ischemic stroke and PFO is three times higher than siblings of patients without PFO [8].

**Figure 1:**
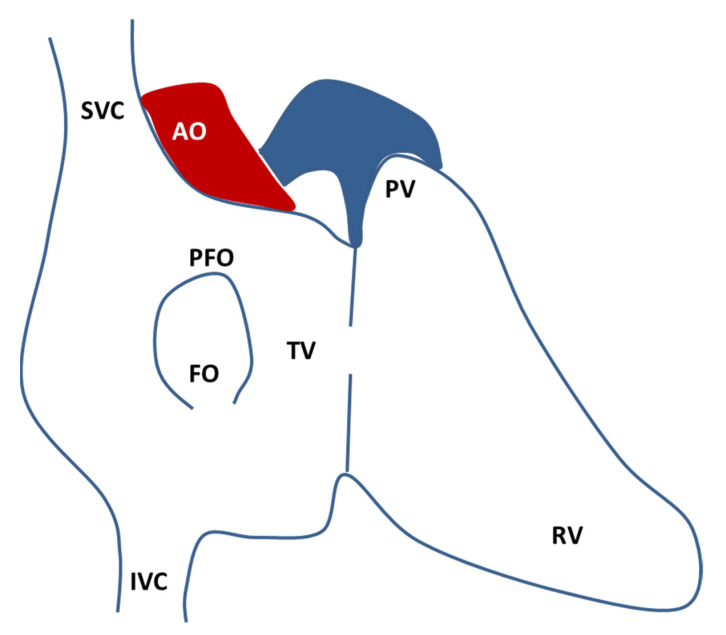
Schematic drawing of the atrial septal anatomy seen from the right atrium (redrawn from Calvert, P., Rana, B., Kydd, A. et al. Patent foramen ovale: anatomy, outcomes, and closure. Nat Rev Cardiol 8, 148–160 (2011). https://doi.org/10.1038/nrcardio.2010.224 accessed date 28 October 2021.). The fossa ovalis (FO) is composed by the septum primum and the septum secundum and the usual location of the PFO is on the anterosuperior border of the FO. AO, aorta; FO, fossa ovalis; IVC, inferior vena cava; PFO, patent foramen ovale; PV, pulmonary valve; RV, right ventricle; SVC, superior vena cava; TV, tricuspid valve.

PFO (Figure 1) is the result of an incomplete postnatal merger of primary and secondary atrial septum. The requirement for two different atrial septa and the high patency rate indicate how atrial septation is a protracted and complex process [9]. In week 4 of human gestation, a mesenchymal structure (the primary septum) arises from the atrial roof, shaping a primary foramen that gradually closes. Then, in week 5, a secondary foramen is formed by the coalescence of perforations of the primary septum. Around week 12, the right atrial roof folds downward and gradually become the secondary septum. After birth, the decreased pulmonary resistance and the increased pressure in the left atrium force the primary septum against the secondary septum: the structures merge, the foramen closes, and the remnant on the right atrium is called fossa ovalis [9]. The need for right-to-left shunt throughout fetal life, particularly during the prolonged gestations of mammals, must be balanced with the progressive septation process. It has been proposed that a mechanism unbalanced towards an incomplete septation could provide greater reproductive success, at the expense of negligible impact in post-natal life [9].

The complexity of the atrial septation process is reflected by the intricacy of the involved genetic pathways. The highly conserved Notch signaling pathway is implicated in heart development, with a clear role in Foramen Ovale closure [10,11]. Other potentially involved genes (GATA Binding Protein 4, *GATA4*; T-Box Transcription Factor 20, *TBX20*; NK2 Homeobox 5, *NKX2-5*; Zic Family Member 3, *ZIC3*) were initially recognized as pathogenic for Atrial Septal Defects (ASD) [12,13]. Co-existence of other atrial abnormalities, such as right atrial septal pouch, Eustachian valve and Chiari’s network, has been reported as added factors in increasing PFO-related stroke risk [14,15]. There are no data in the literature about the inheritance of these minor atrial abnormalities neither individually nor in association with PFO. Similarly, the inheritance of the atrial septal aneurysm (ASA) is unknown.

Given the high rate of PFO in the general population, the large majority of PFOs do not play any pathological role. For those PFO subjects that suffer a cryptogenic ischemic stroke, the role of PFO as a contributing factor should be considered along with clotting disorders that may increase stroke risk. The mechanisms through which PFO may contribute to the risk of stroke and systemic embolism are at least two [16]: acting as a channel for paradoxical embolism (from a travelling venous clot, particularly in presence of a prothrombotic state, or from a clot formed in situ within the PFO) and triggering atrial arrhythmias because of electrical signaling disruption (the latter has been hypothesized especially in the setting of PFO associated with ASA or a hypermobile atrial septum). In both situations, the pattern of ischemic lesion on neuroimaging studies is coherent with an embolic source [17,18] and the potential genetic link between the presence of ASD, including PFO, and atrial arrhythmias, including atrial fibrillation, has not been extensively explored to date. The increasing knowledge has led to the proposal of an updated nomenclature and classification of potential causative mechanisms of embolic stroke in patients with a PFO [19].

Despite PFO being intensively studied for therapeutic approaches, little is known about its pathophysiological role. Can PFO be considered as a benign anatomical variant, or does it gather a more variegated range of alterations, some of which at a greater risk of comorbidity? Genetic findings may elucidate the genesis and significance of PFO.

The aim of this review is to evaluate the contribution of genetic alterations in PFO development. We systematically reviewed studies assessing rare and common variants in subjects with PFO.

## 2. Materials and Methods

### 2.1. Sources

This systematic review follows the Meta-Analyses and Systematic Reviews of Observational Studies (MOOSE) group guidelines [20]. The study protocol was registered with PROSPERO.

We searched PubMed, EMBASE, Cochrane Central and Medrxiv databases for studies addressing genetic analysis in PFO subjects published within 30th June 2021. We used the following keywords: PFO OR “patent foramen ovale” AND (genetic OR gene OR mutation OR polymorphism). In addition, we applied forward and backward citation tracking to improve the results.

### 2.2. Eligibility Criteria

All studies presenting original data that reported mutation or variation frequencies in subjects with PFO were included. We limited the selection to English-language studies and excluded case reports and studies on nonhuman subjects. We excluded studies evaluating genetic alterations in Atrial Septal Defects (ASD) or in which PFO was not a per se group but was included in broader clusters of congenital malformations. Abstracts presented at relevant scientific meetings were included if reported data fulfilled sufficient completeness criteria. Studies reporting the same dataset were excluded.

We relied on the diagnostics put in place in the original study for PFO definition. No limitation was implemented according to type of ascertainment technique or PFO grading. 

Two investigators (MP, CV) independently screened the identified literature and selected studies according to the abovementioned criteria.

### 2.3. Data Extraction

The NIH Quality Assessment Tool for Observational Cohort and Cross-Sectional Studies was applied to each eligible publication. We then extracted the following information: authors, year of publication, country of enrollment, study design, population characteristics, evaluated gene(s) with the frequency of mutations or variations. When possible, missing values were computed as shown elsewhere [21]. Disagreements between the two reviewers were resolved by consensus.

## 3. Results

**Figure 2:**
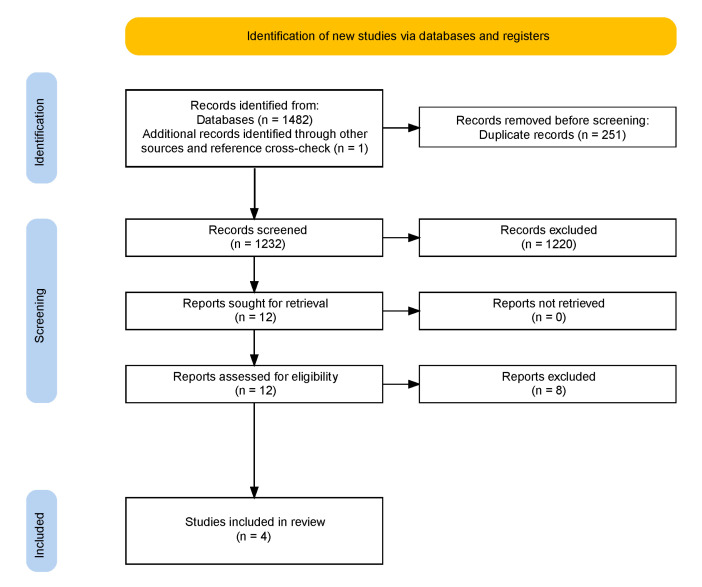
PRISMA flow chart.

We identified 1231 studies. From those, we screened 12 publications. Four of them were included in the review (Figure 2).

According to the NIH Quality Assessment Tool for Observational Cohort and Cross-Sectional, all studies achieved a fair level of quality.

Baseline demographics and relevant clinical data of the four included studies are shown in Table 1, while genetic analyses are shown in Table 2. Two of the four studies considered evaluated the coding sequence of the *NKX2-5* gene [22,23], while the remaining assessed the presence of two specific variants (rs2200733 and rs10033464) located in chromosome 4q25 [24] and the *GATA4* p.S377G variant [25], respectively. All studies but one [23] evaluated PFO through TEE. However, in Elliott et al., 2003 [23] the diagnosis of PFO was proven, since all PFO patients underwent a closure procedure. 

The prevalence of variants in subjects with PFO ranged from 3 to 62%, with the highest reached in studies enrolling people with stroke or TIA (Table 2). The minor allele frequencies (MAF) of the variants under analysis are also reported (https://gnomad.broadinstitute.org/, accessed on 28 October 2021). Only the rs2200733 variation of chromosome 4q25 was significantly associated with PFO in atrial fibrillation (AF) patients (OR 0.610, 95% CI 0.378–0.984, *p* = 0.043) [24].

## 4. Discussion

Our systematic review only found four studies evaluating genetic anomalies in PFO subjects. All of them included patients with PFO and either paradoxical embolism, stroke or TIA, or AF. This last association deserves a dedicated reasoning. While isolated PFO is usually not associated with atrial arrhythmias, these can be triggered by ASA or hypermobile atrial septum, often in combination with a PFO [26]. The underlying hypothesis is that ASDs induce atrial vulnerability, i.e., the electrophysiological trend to induce AF. Indeed, the rate of AF or atrial flutter in patients with PFO and/or ASA is 20–42% [27] and inducible AF longer than 60 s was documented in 58% of patients with PFO and/or ASA, as compared to 25% of patients without. A proposed mechanism is the stretch or pressure on the atrial septum [28].

**Figure 3:**
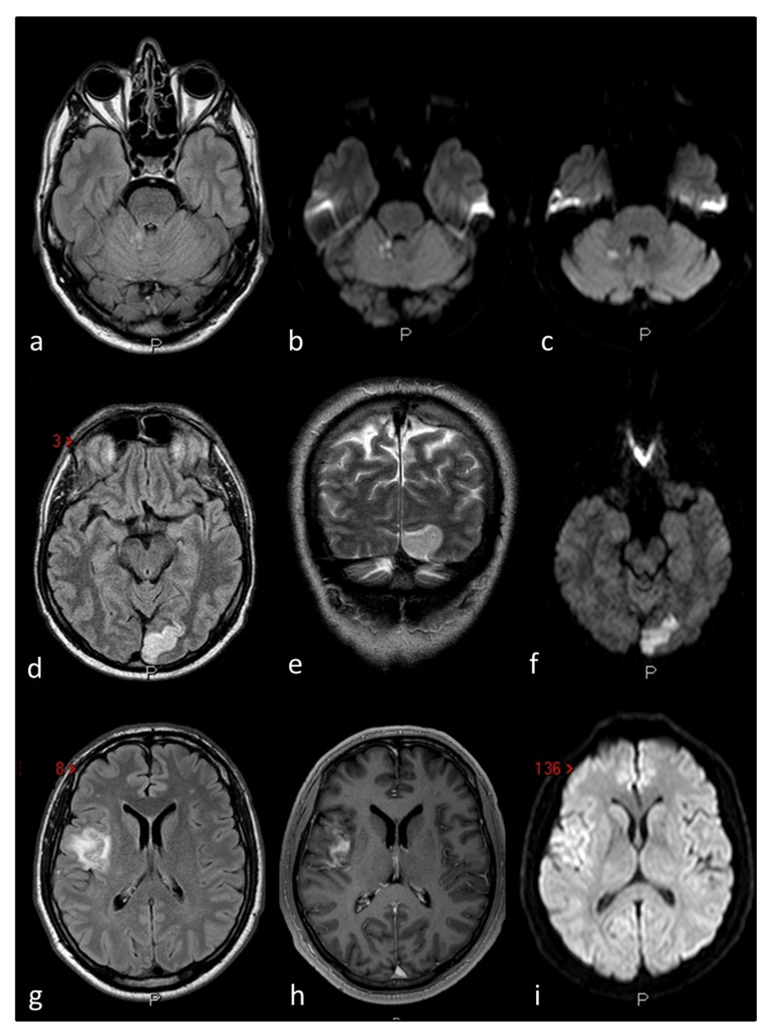
The figure outlines three examples of cryptogenic embolism in patients with PFO as imaged in Magnetic Resonance Imaging (MRI) of the brain: Patient 1 (**a**–**c**) multiple right cerebellar recent ischemic lesions on axial FLAIR (**a**) and DWI (**b**,**c**) MRI sequences. Patient 2 (**d**–**f**) left occipital ischemic stroke in a patient with migraine with aura and PFO on axial FLAIR (**d**) coronal T2W (**e**) and DWI MRI sequences. Patient 3 (**g**–**i**) right temporo-parietal ischemic stroke on axial FLAIR (**g**) contrast-enhanced axial T1W (**h**) and DWI (**i**) MRI sequences.

The included studies are quite heterogeneous in terms of population, intervention, and sample size. Two of them respectively evaluated 25 [23] and 100 subjects [22], and thus their sample size is probably inadequate. We did not find any genetic variant or mutation associated with PFO, except for the rs2200733 variation of chromosome 4q25 in AF patients. The rs2200733 variation, lying in an intergenic genomic region, is indeed a known risk factor for AF [29]. The closest gene to this variation is Paired Like Homeodomain 2 (*PITX2*), implicated in cardiac morphogenesis, particularly in the differential identity of left and right atria [30,31]. The AF risk of rs2200733 variant and PITX2 absence, as seen in knockout mice models [32,33], is probably due to alterations of the sinoatrial node formation. However, to our knowledge, no other studies investigated the role of the rs2200733 variant nor the *PITX2* gene in foramen ovale closure. On the other hand, non-PFO studies have also been reported in the literature but none of them found a positive association between the variant and the diseases investigated [34,35]. The reported association between PFO and the rs2200733 variant in patients with AF requires further validation as it was described in a single study published as a conference abstract [24]. Therefore, the hypothesis of a genetic link between PFO and AF may be proposed, enriching the list of potential correlations between PFO and stroke risk from which derive considerations with important therapeutic implications. This consideration could be particularly of interest in terms of practical translation because the main reason for the search for a PFO is a clinical presentation with an ischemic stroke with an imaging pattern suggestive of embolism (Figure 3) [36], which is defined as cryptogenic mainly by virtue of the exclusion of causes (atherothrombotic and AF) and substantially falls within the concept of Embolic Stroke of Unknown Source (ESUS). The possibility of testing AF in patients with ESUS strictly depends on the modalities and duration of cardiac monitoring and remains a matter of debate despite the negative result of therapeutic trials with direct oral anticoagulants, including the subgroup of patients with PFO [37].

Despite the lack of epidemiological studies and the scarceness of evidence so far, animal studies and other studies that did not fulfil the inclusion criteria of the review indicate a robust genetic background in PFO.

A gain-of-function mutation in the *TBX20* gene (p.I121M) was found in a patient with ostium secundum atrial septal defects (ASDII) and in his sister and mother, both affected by large PFOs [12]. Similarly, the mother of a proband with an ASD, both carrying such mutation, had a large PFO [38]. TBX20 is a T-box transcription factor, and its gain-of-function significantly enhanced transcriptional activity, which was further increased in the presence of its cardiac co-transcription factors GATA4/5 and NKX2-5. The homeobox transcription factor *NKX2-5* mutations have been associated, among others, with ASD [39]. *NKX2-5* heterozygous-null mice showed a significant 2.5–3.5 higher rate of PFO compared to wild type mice [40]. The synergistic role of *TBX20* and *NKX2-5* has been demonstrated in an animal study in which the rate of PFO was more than double in mice defective for *TBX20* or *NKX2-5* compared to wild type mice. Interestingly, mice defective for both genes showed a higher rate of complex PFO (PFO + atrial septal aneurysm) or even ASD [41].

GATA4 is a zinc finger transcription factor that, similarly, has a synergistic interaction with TBX20 and, likewise, mutations are responsible for Congenital Heart Defects (CHD), particularly ASD [42]. The interaction between the cardiac T-boxes (TBX20 and TBX5) and the transcription factors NKX2-5 and GATA4 suggest the complexity of the genetic regulation of cardiac septal formation.

**Figure 4:**
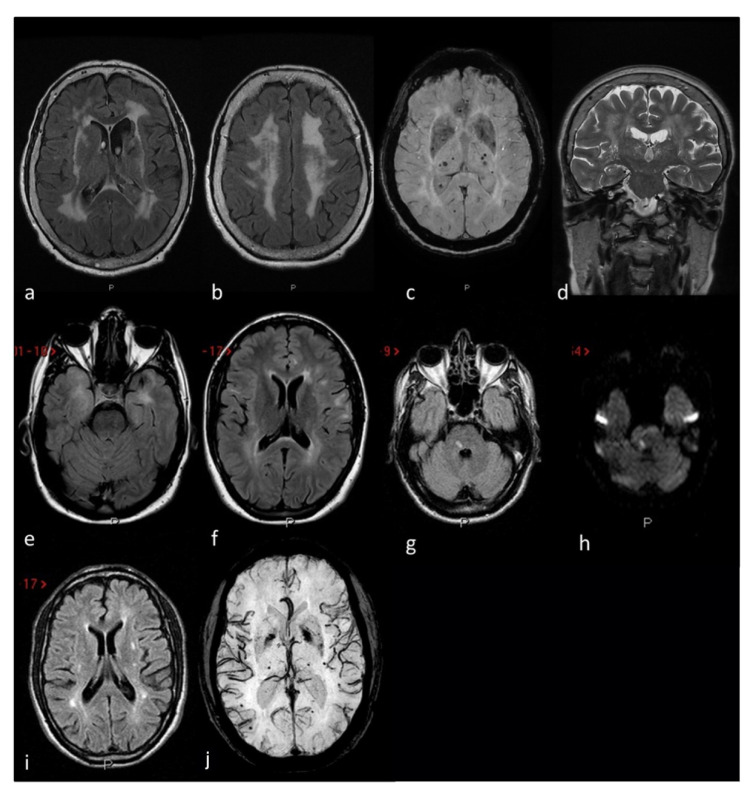
MRI markers of small vessel disease in three CADASIL patients: Patient 1 (**a**–**d**) severe leukoaraiosis involving subcortical white matter, external capsule and periventricular regions in a symmetrical pattern on axial FLAIR (**a**,**b**) MRI, associated with deel and lobar supratentorial microbleeds on SWI (axial MiP sequences) MRI (**c**) and enlarged perivascular spaces in the basal ganglia on coronal T2W (**d**) MRI. Patient 2 (**e**,**f**) anterior temporal lobe involvement (**e**) and periventricular and iuxtacortical white matter hyperintensities (**f**) on axial FLAIR MRI. Patient 3 (**g**–**j**) recent subcortical ischemic stroke on the right portion of the pons on FLAIR (**g**) and DWI (**h**) MRI sequences, supratentorial white matter hyperintensities with a dotted distribution involving the external capsule and the periventricular regions on axial FLAIR (**i**) MRI and multiple supratentorial deep and lobar microbleeds on SWI (MiP sequences) MRI (**j**).

Another signaling pathway to consider is Notch. Animal studies demonstrated its important role in the endothelial-to-mesenchymal transition (EndMT) occurring during the Foramen Ovale closure [10]. EndMT is an important mechanism involved in heart septation. The Notch receptors on the cell surface induce EndMT by activating the transcription factor Snail. In mammals, there are four types of Notch receptor (Notch 1–4) and five ligands (Delta-like-1, −3 and −4, and Jagged−1 and −2). Neurogenic locus notch homolog protein 3 (*NOTCH3*) is the leading gene in this process. Indeed, in rat hearts, increasingly high levels of Notch3 receptor expression, and to a lesser extent Notch1 and Jagged-1, were found in the foramen ovale region during its closure process [10]. The EndMT converted the cells to a fibroblast-like phenotype, leading to the merge of the tissues. Interestingly, *NOTCH3* mutations are responsible for CADASIL (Cerebral Autosomal Dominant Arteriopathy with Subcortical Infarcts and Leukoencephalopathy), the most common genetic cause of small vessel disease ischemic stroke, often associated with migraine with aura. The main pattern of cerebrovascular damage in CADASIL is small vessel disease, as seen in Figure 4. The association of PFO with CADASIL has been reported but remains controversial [43,44] and needs further insights. As of human studies, exome sequencing of members of a family with a dominant inheritance of ASD, including PFO, identified the pathogenic variant of the Notch homolog 1, translocation-associated (Drosophila) (*NOTCH1*) gene (c.3835C>T) [45]. However, only a minor decrease in signaling activity was found.

So far, no single genetic alteration responsible for PFO has been found. Cardiac development, particularly atrial septation, may be influenced by the combination of different genetic factors rather than by a single gene variant. In this light, a recent genome-wide association study on Congenital Heart Defects (CHD) identified 20 risk-inducing SNPs [46]. Knockout mice for the related genes, however, did not show clinical CHD, reinforcing the concept of a multigenic etiology of the defects.

## 5. Conclusions

Research on PFO is mainly focused on the best therapeutical approach in the setting of paradoxical embolism. Fewer efforts have been made on the research on the pathogenesis of this common condition. The results of this review highlight the need for broadening the research on physiopathology and, more specifically, on the genetics of PFO. We believe that further insight from genetic studies may help to better evaluate at the single-patient level the incidental or co-causative role of PFO in the setting of cryptogenic stroke, potential correlation with migraine, and potential value for genetic screening of at-risk first-degree relatives.

## Figures and Tables

**Table 1:** Study and patient characteristics of the included studies.

Author, Year, Country of Patients	Study Design	Genotyping Method	Population	Sample Size	Mean Age (Years, SD)	Male (%)
Belvis, 2009, Spain [22]	case–control	gene specific amplification and sequencing	Stroke/TIA patients with or without PFO	100	56.5 (12.4)	58%
Bollmann, 2010, Germany [24]	case–control	commercial real-time PCR for specific SNP + FRET	Atrial fibrillation patients with or without PFO	508	57 (10)	70%
Elliott, 2003, Australia [23]	cohort	gene specific amplification and sequencing	PFO with paradoxical embolism which underwent percutaneous closure	25	48.7 (15.3)	48%
Marjaneh, 2011, Australia & Germany [25]	case–control	gene specific amplification and sequencing commercial genotyping for specific SNP	PFO (with or without stroke/TIA) vs. controls	752	58.7 (12.2)	54.8%

PCR = polymerase chain reaction; SNP = single nucleotide polymorphism; FRET = fluorescence resonance energy transfer.

**Table 2:** Results of the included studies.

*NKX2-5*		Allelic Frequency		
Belvis, 2009 [22]	c.172A > G ^1^ Glu21 =	found in the 36% of healthy controls (30%AG and 6%GG) (G = 4.11e^−1^ *)	Stroke with PFO: 21/34 (62%) vs. Stroke without PFO: 33/66 (50%)	*p* = 0.295
	c.182C > T ^1^ Arg25Cys	not found in 100 screened alleles from healthy controls (T = 4.14e^−3^ *)	Stroke with PFO: 0/34 (0%) vs. Stroke without PFO: 2/66 (3%)	*p* = 0.547
	c.2357G > A ^1^	not found in 100 screened alleles from healthy controls	Stroke with PFO: 1/34 (3%) vs. Stroke without PFO: 0/66 (0%)	*p* = 0.340
	c.2850C > A ^1^	found in the 60% of healthy controls (40%AC and 20%AA)	Stroke with PFO: 19/34 (56%) vs. Stroke without PFO: 35/66 (53%)	*p* = 0.835
Elliott, 2003 [23]	-	-	No mutations found in PFO patients	
Chromosome 4q25				
Bollmann, 2010 [24]	chr4:110789013C > T rs2200733 rs10033464	T = 0.184 *	AF without PFO vs. AF with PFO: OR 0.610, 95% CI 0.378–0.984 No association with PFO	*p* = 0.043
***GATA4***				
Marjaneh, 2011 [25]	c.1647A > G Ser377Gly ^1^	G = 0.104 *	PFO (with or without stroke/TIA): 46/183 (25%) vs. controls: 73/340 (21%)	*p* = 0.340

^1^ variants are reported as described in the respective articles and indicated pooling heterozygous and homozygous. AF: atrial fibrillation; * minor allele frequency (MAF) (https://gnomad.broadinstitute.org/ accessed date 25 November 2021).
